# An Effective Low-Cost Remote Sensing Approach to Reconstruct the Long-Term and Dense Time Series of Area and Storage Variations for Large Lakes

**DOI:** 10.3390/s19194247

**Published:** 2019-09-30

**Authors:** Shuangxiao Luo, Chunqiao Song, Kai Liu, Linghong Ke, Ronghua Ma

**Affiliations:** 1 Key Laboratory of Watershed Geographic Sciences, Nanjing Institute of Geography and Limnology, Chinese Academy of Sciences, Nanjing 210008, China; lushxidyx@163.com (S.L.); kliu@niglas.ac.cn (K.L.); rhma@niglas.ac.cn (R.M.); 2 University of Chinese Academy of Sciences, Beijing 100049, China; 3 School of Earth Sciences and Engineering, Hohai University, Nanjing 211100, China; kelinghong@hhu.edu.cn

**Keywords:** remote sensing, large lakes mapping, water occurrence frequency (WOF), google earth engine, Lake changes

## Abstract

Inland lakes are essential components of hydrological and biogeochemical water cycles, as well as indispensable water resources for human beings. To derive the long-term and continuous trajectory of lake inundation area changes is increasingly significant. Since it helps to understand how they function in the global water cycle and how they are impacted by climate change and human activities. Employing optical satellite images, as an important means of lake mapping, has been widely used in the monitoring of lakes. It is well known that one of the obvious difficulties of traditional remote sensing-based mapping methods lies in the tremendous labor and computing costs for delineating the large lakes (e.g., Caspian Sea). In this study, a novel approach of reconstructing long-term and high-frequency time series of inundation areas of large lakes is proposed. The general idea of this method is to obtain the lake inundation area at any specific observation date by referring to the mapping relationship of the water occurrence frequency (WOF) of the selected shoreline segment at relatively slight terrains and lake areas based on the pre-established lookup table. The lookup table to map the links of the WOF and lake areas is derived from the Joint Research Centre (JRC)Global Surface Water (GSW) dataset accessed in Google Earth Engine (GEE). We select five large lakes worldwide to reconstruct their long time series (1984–2018) of inundation areas using this method. The time series of lake volume variation are analyzed, and the qualitative investigations of these lake changes are eventually discussed by referring to previous studies. The results based on the case of North Aral Sea show that the mean relative error between estimated area and actually mapped value is about 0.85%. The mean R^2^ of all the five lakes is 0.746, which indicates that the proposed method can produce the robust estimates of area time series for these large lakes. This research sheds new light on mapping large lakes at considerably deducted time and labor costs, and be effectively applicable in other large lakes in regional and global scales.

## 1. Introduction

Inland lakes play a crucial role in global water cycle. Monitoring the physical characteristics and dimensions of lakes is rather crucial to study how they are impacted by climate change and human activities [1,2]. This was demonstrated in a number of studies using remote sensing to capture the spatial and temporal variations of lakes globally [3,4]. Pekel et al. [5] quantify the changes in global surface water (GSW) between 1984 and 2015 at 30-m resolution by using three million Landsat satellite images. They record the months and years in which water is present, the changes occur, and changes in seasonality and persistence. On the basis of these results they concluded that permanent surface water has disappeared from an area of almost 90,000 km^2^ over the past 32 years and about 70% global net permanent water loss occurred in the Middle East and Central Asia linked to drought and human actions. Zou et al. [6] detected open-surface water bodies in all Landsat 5, 7, and eight images (~370,000 images, >200 TB) of the CONUS and generated 30-m annual water body frequency maps for 1984–2016. They analyzed the inter-annual variations and trends of year-long water body area, examined the impacts of climatic and anthropogenic drivers on water body area dynamics, and explored the relationships between water body area and land water storage. Their results constitute a valuable source of data on lake abundance and size distribution. In summary, the predecessors have done a lot of significant work of monitoring spatial-temporal changes of inland waterbodies in large scale and long term.

One of the most significant difficulties in extracting inland water body, particularly for the long time-series task, lies in the immense costs of labor consuming and computing resource required for mapping large lakes. For example, Caspian Sea covers more than thirty Landsat imagery tiles for mapping one-time of the entire lake inundation area. However, there is little published information on solving the difficulty of collecting complete cloud-free remote sensing images covering the entire large lake within one observation period, and reducing the heavy labor and computing workload for mapping these large lakes [7]. Therefore, the new method for extracting the entire inundation areas of large lakes for any pre-specified observation date that only needs one image scene is proposed in this study. The new method is different from the traditional remote sensing method in image extraction, avoiding the difficulty of data missing and poor image quality. This method not only reduces the dependence on the quality of remote sensing image, but also greatly reduces the consumption of manpower and time. The key idea of this method originates from the fact that the inundation area variation of these large lakes can be better monitored in the lakeshore sites with relatively slighter terrain slopes. Hence, we can focus on delineating the evolution trajectory of these lake shorelines which have obvious shifts reflected in time series of remote sensing image.

Owing to the establishment of the GEE platform and the release of the GSW dataset, this idea can be efficiently conducted based on the multi-year (1984–2015) water inundation frequency map of these lakes. We select the relatively flatter lake shores as study sites by referring to the water inundation frequency map. We then generate the frequency-area look-up table through establishing the regression relationship between the average water frequency of shoreline segment and corresponding lake inundation area. Thus, the lake inundation area at any given observation date can be reconstructed by only extracting the shoreline segment of selected lakeshore sites. In further step, the time series of reconstructed lake area will be compared with the time series of water level to simply evaluate the reconstruction performance.

The remainder of the manuscript is structured as follows. In Section 2, specific study sites of five lake cases, Lake Athabasca, Caspian Sea, North Aral Sea, Lake Balkhash, and Lake Issyk-Kul, are described. In Section 3, the used data and pre-processing procedures are introduced. In Section 4, the methodology of generating lookup table of water inundation frequency, reconstructing time series of lake areas, evaluating estimated areas, water level and volume variation are explained. The analysis of all experimental results, discussions and summary of this study are presented in Section 5 and Section 6, respectively.

## 2. Lake Cases Selected in This Study

**Figure 1:**
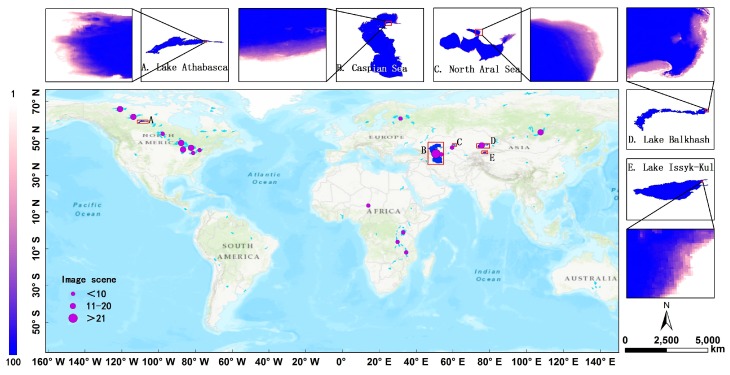
Geographical distribution of the five selected lakes including: (**A**) Lake Athabasca, (**B**) Caspian Sea, (**C**) North Aral Sea, (**D**) Lake Balkhash, and (**E**) Lake Issyk-Kul.

It is well accepted that the imagery acquisition for these great lakes is a heavy and inefficient task. We screened there are 18 large lakes with area more than 10,000 km^2^ around the world and counted the total number of Landsat image scenes covering these lakes (Figure 1). It confirms the difficulty for us to obtain a complete and temporal-uniform image coverage of these large lakes. There are 8 lakes requiring more than ten Landsat tiles to retrieve one-time of inundation area. We select five lakes located in North America and Eurasia, including Lake Athabasca, Caspian Sea, North Aral Sea, Lake Balkhash and Lake Issyk-Kul, as study cases to demonstrate how to obtain long time series of inundation areas of these large lakes more efficiently based on our proposed method.

Lake Athabasca, straddling the provinces of Alberta and Saskatchewan, is the fourth largest lake over northern Canada (58°35′31′′ N–59°37′37′′ N, 106°30′29′′ W–111°11′59′′ W). It covers a surface area of 7800 km^2^ [8]. As shown in Figure 1, we chose a study area of approximately 20 km^2^ in the eastern part of Lake Athabasca in 20-km west of Canadian city Fond-du-Lac. The terrain of the study region is relatively flat and its lake water depth is shallow. It can sensitively reflect the lake area changes with the water level shifts.

Caspian Sea (36°34′5′′ N–47°8′51′′ N, 46°43′58′′E–54°4′21′′E) is bordered by the states of Azerbaijan, Federation of Russia, Islamic Republic of Iran, Kazakhstan, and Turkmenistan [9]. It is the largest inland lake on Earth, with an area of 384,400 km^2^, a volume of 78,700 km^3^ and a length over 1200 km from north to south [10]. Caspian Sea is 27 m below the global mean sea level and is conventionally comprised by three parts: southern, middle and northern Caspian [11]. The southern basin is more than 1000 m deep, while the northern basin is shallow with a depth between 1 and 10–15 m [12]. The bottom of the North Caspian is a wave-like sedimentary plain with only 1% volume of the total water volume. In this study, we chose a study area of approximately 1169 km^2^ in north-eastern part of the northern basin. Similarly, the lakeshore slope of the focused region is gentle and can reflect the changes of water level and area sensitively.

Aral Sea (center of 45°N, 59°E) is a terminal (closed basin) lake lying in the vast Kara Kum (Red Desert) and Kyzyl Kum (Black Desert) of Central Asia [13]. Its drainage basin encompasses more than 2 million km^2^ within seven nations (Uzbekistan, Turkmenistan, Kazakhstan, Afghanistan, Tajikistan, and Iran). Covering a surface area of 67,500 km^2^ in 1960, the Aral Sea was the world’s fourth largest inland water body in area [14]. Due to human overuse and climate variability, it shrank dramatically and had separated into four separate water bodies by September 2009. The maximum water level decline was more than 26 m, whereas the lake surface area decreased by 88% and the water volume by 92%. The lake salinity increased by more than 20 folds. In our study, we chose a study area of approximately 176 km^2^ at the northernmost part of North Aral Sea with slight terrain slope (46°07′20′′ N–46°47′08′′ N, 59°58′34′′ E–61°32′51′′E).

Lake Balkhash is an endorheic saline lake in the south-west of the Republic of Kazakhstan (45°02′54′′ N–46°50′04′′ N, 73°23′48′′E–79°14′54′′E). It is the fourth longest lake over the world, with a length of 605 km from east to west [15]. The Lake volume is 111.50 km^3^ and the mean depth is 9 m. The lake surface area is approximately 18,000 km^2^ [16]. The lake catchment is divided into south-western and eastern basin. It shows a clear salinity gradient, with its south-western basin having a salinity of <2 g·L^−1^ whereas eastern basin having a salinity of 4 g·L^−1^. As shown in Figure 1, we select a study area of about 160 km^2^ at the easternmost part of Lake Balkhash.

Lake Issyk-Kul is located in a closed basin at an altitude of 1606 m in northwest of the Tien Shan Mountains in the Central Asia (42°09′04′′ N–42°46′16′′ N, 76°10′58′′E– 78°19′41′′E). It is one of the largest lakes of the world with a water volume of 1730 km^3^ and a surface area of 6247 km^2^ [17]. The western lake shore near Balykchy receives 120 mm/yr of rainfall, while the eastern lake shore near Karakol receives 720 mm/yr [18]. Despite maximum depth of Lake Issyk-Kul is 668 m, the lake has large, shallow shelf regions, predominantly in the east and west ends. 27% of the lake area is shallower than 50 m [19]. In our study, we chose a study area of approximately 27 km^2^ at the easternmost part of Lake Issyk-Kul.

## 3. Study Data

### 3.1. The Global Surface Water Dataset for Extracting Monthly Lake Area During 1984–2015

This European Commission’s JRC GSW dataset maps the location and temporal distribution of surface water over a 32-year period (from 16 March 1984 to 10 October 2015) according to 3,066,102 scenes from Landsat 5, 7, and 8 at 30 m resolution. The expert system was developed to assign each pixel to one of three target classes, either water, land or non-valid observations (snow, ice, cloud or sensor-related issues) based on multispectral and multi-temporal attributes [5]. The results were collated into a monthly history for the entire time period and two epochs (1984–1999, 2000–2015) for change detection, and provided statistics on the extent and change of those water surfaces. The monthly water history dataset contains a set of 380 global scale maps documenting the water presence for each month of the 32-year archive. In this study, surface water occurrence and monthly water history range from March 1984 to October 2015 were used. Surface water occurrence contains the frequency with which water was present on the surface from March 1984 to October 2015. Monthly water history, water detection for the month, holds the entire history of water detection on a month-by-month basis. The collection contains 380 images, one for each month between March 1984 and October 2015. However, the monthly data does not cover the complete and temporally consecutive water body boundaries for each month due to the missing high-quality images. The number of available monthly lake shorelines varies with different lakes and years.

### 3.2. Landsat 8 OLI Imagery for Mapping Lake Areas During 2013–2018

Landsat data were downloaded from the U.S. Geological Survey (USGS) Earth Resources Observation and Science Center (EROS) website (source: http://glovis.usgs.gov). At each Landsat tile (path/row individual), we collect Landsat 8 OLI/TIRS C1 Level-1 data products for 6 years from 2013 to 2018 (Table 1). The data were used to obtain the lake inundation areas over targeted lakeshores in 2013–2018. Among them, five dates of Landsat images for North Aral Sea that can cover the entire lake area (two Landsat tile) were mapped to compare with the reconstructed lake area based on the proposed method.

### 3.3. Lake Water-Level Data from Satellite Altimetry

Satellite radar altimetry is originally designed for the purpose of observing ocean surface. The development of satellite altimetry in the last decade has made it possible to continuously monitor inland water levels and generate long-term time series of lake stages. Owing to the efforts of the Laboratoire d’Etudes en Géophysique et Océanographie Spatiales (LEGOS) altimetry datasets [20,21], a web database (HYDROWEB: http://www.LEGOS.obs-mip.fr/soa/hydrologie/HYDROWEB) containing time series over water levels of large rivers, lakes and wetlands on a global scale has been developed over the past five years. Therefore, water levels of the five lakes in our study are based on the merged LEGOS altimetry datasets (including Topex/Poseidon (T/P), Jason-1/2, GFO, ENVISAT data), Jason-3, SARAL and Sentinel-3A data (shown in Table 2). The track numbers of different altimeter satellites for each lake are shown in Table 3. Combining different altimetry missions not only increases the spatial coverage of altimeter satellites, but also extends the temporal span to nearly 20 years [22]. Crétaux [23] has introduced the main processing steps of synthesizing the lake level series from different satellite missions covering the same lake: firstly, each satellite data is processed independently; then T/P data is used as a reference to minimize potential radar instrument deviations between different satellites; finally, lake levels from the different satellites are merged on a monthly basis. This method can improve the consistency and precision of lake level data derived from the multi-satellite observations [21].

In this study, the time series of water level in North Aral Sea range from October 1992 to September 2016, and water level data for other four lakes cover the period from September 1992 to October 2018. Water level data of the five lakes are all post-processed to the monthly means of all measurements within one-month time window.

## 4. Methodology

### 4.1. Reconstructing Time Series of Inundation Areas of Large Lakes

**Figure 2:**
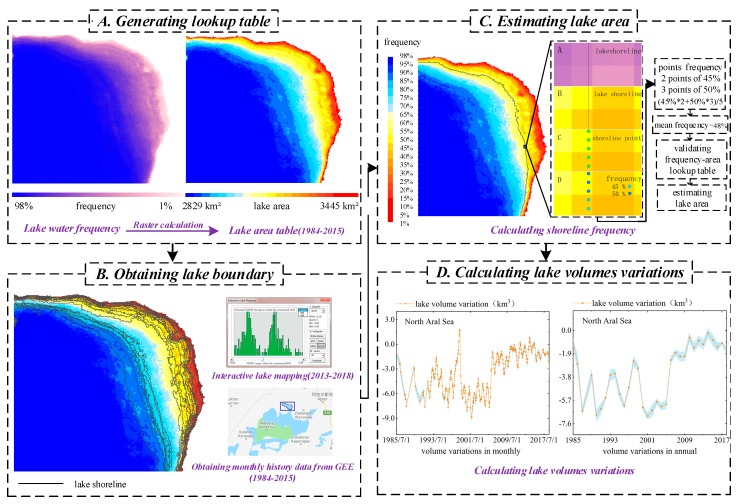
Workflow of the method proposed for reconstructing time series of lake area and volume variations including: (**A**) generating lookup tables, (**B**) extracting lakes boundary, (**C**) estimating lake areas, and (**D**) calculating lake volumes variations.

Our method for reconstructing lake surface area includes four steps as described in the sub-sections below (see Figure 2). First, we select one lakeshore segment for each lake with gentle bathymetry terrains as target area, which tends to show more obvious lake shoreline shifts with water level changes, as introduced in Section 2. Then, we generate area-frequency lookup-tables by performing a raster calculation on the water frequency map of the GSW dataset. After acquiring the lake shoreline section within the defined target area in any specified observation date, we calculate the spatially averaged water frequency value by overlapping the shoreline on the GSW occurrence map and further estimate the lake area via frequency-area lookup-table. Finally, the water level time series are used to evaluate the performance of the reconstructed lake area variations.

#### 4.1.1. Generating Lookup Tables of Shoreline Sater Frequency and Corresponding Lake Area

According to JRC GSW occurrence maps (1984–2015), we selected lake shores with more significant and gradient changes in water frequency as the target area. The lake water frequency higher than or equal to 98% is classified as permanent water bodies. The rest are divided into about 20 grades by the frequency interval of 5%. In order to calculate the sensitivity of the water frequency grade determination, we also calculated the relative standard deviation under the grading interval of 2.5% and 7.5% (Figure S1 in supplementary information). Taking North Aral Sea in the can be referred in Table S1 as an example, the experimental results showed that the estimation results of the grading intervals as 2.5%, 5% or 7.5% are basically consistent. In order to facilitate the calculation, the interval of 5% was selected as the grading interval value for the following experiment analyses. We calculate the water body area of each grade and generate the area-frequency lookup tables for each lake.

#### 4.1.2. Extracting Lakes Shorelines from Monthly GSW Dataset and OLI Imagery

Monthly GSW data (1984–2015) and Landsat 8 OLI/TIRS C1 Level-1 products (2013–2018) are combined as a 35-year (1984–2018) dataset for reconstructing long-term lake inundation area variations. The processing procedures of the lake extraction from the two datasets are introduced as follows:

Monthly surface water area data of GSW dataset from 1984 to 2015 can be downloaded from GEE. The monthly lake inundation data is further screened manually and the data with incomplete boundaries or obvious noisy shifts are removed. Although 320 months of data are available on GEE, it was found that not all months of our study area is usable in actual operations after the manual inspection.

Level-1 Landsat data images during 2013–2018, available in study areas, are all downloaded from USGS. The method of extracting water inundation areas via a two-step NDWI (Normalized Difference Water Index) threshold segmentation scheme was applied in this study [24]. In the first step, all potential water body pixels are filtered by a loose initial threshold segmentation based on calculated NDWI maps of selected multiple spectral images. The threshold is set to NDWI > –0.1, which is a conservative estimate to mask out various water bodies in different spectral properties at global scale; the second step is locally iterative segmentation for each potential lake entity, and whose goal for obtaining stable lake boundary via consecutive segmentations with the least shifted water area. The NDWI, generated by the normalized water index between the green (ρgreen) and near infrared (ρNIR) spectral bands, is proposed by McFeeters [25]. Moreover, mapping accuracy of water body boundaries is guaranteed by additional visual inspection and manual editing. Compared to the single-threshold segmentation method which uses a fixed threshold to delineate outlines of different lakes with obviously distinctive spectral features, this method can efficiently improve the accuracy of water body extent delineation [26]. Eventually about 101–127 months of lake shorelines can be used for the five lakes via two post-processing methods and the specific information can be referred in Table S2 of supplementary information.

#### 4.1.3. Estimating Lake Areas via Shoreline Water Frequency from Lookup Tables

The completeness of the predefined lake shoreline is closely related to the accuracy of the reconstructed lake area based on lookup table. However, not all of extracted lake shorelines are complete due to imagery quality and mapping error. In order to improve the area reconstruction accuracy, we manually screened the lakeshores before retrieving their overlapped water frequency values, and further filtered the lakeshores in the areas with flat terrain and obvious changes. Then we convert the extracted lake shoreline into a continuous finite number of points and calculate the number of points that fall on the grades representing different water inundation frequencies. Finally, we obtain a mean value of all the points to represent the water frequency of lake shorelines at the specific observation date, which can be further converted to the lake area based on the statistical regression relationship between the shoreline water frequency and lake area in our established lookup table.

#### 4.1.4. Accuracy Assessment of Reconstructed Lake Area Time Aeries

Two methods were used to evaluate the performances of the reconstructed lake areas. We consider the change in lake area is closely related to the water level variations, so we first compared the reconstructed lake areas with water levels from the altimetry satellites. The second method is to directly compare with mapped areas from Landsat imagery for the North Aral Sea with relatively small area. Monthly mean values of lake water level are used for evaluating the estimated area values. We pick the area and water levels of lakes in the same period and calculate the correlation with R^2^ values. R^2^ values is a coefficient of determination, which represents the ratio of the sum of squares caused by X in the sum of squares of Y. In the unary regression analysis, the closer R^2^ values is to 1, the higher reference value of the related equation; on the contrary, the closer to 0, the lower reference value:

(1) $$\bar{y}=\frac{1}{n}\overset{n}{\underset{i=1}{\sum }}y_{i}$$

(2) $$SS_{tot}=\underset{i}{\sum }{\left(y_{i}-\bar{y}\right)}^{2}$$

(3) $$\;SS_{res}=\underset{i}{\sum }{\left(y_{i}-f_{i}\right)}^{2}$$

(4) $$R^{2}=1-\frac{SS_{res}}{SS_{tot}}$$

where $\bar{y}$, $SS_{tot}\;,\;SS_{res}$ are the mean value of y, sum of Squares for total and sum of squares for error. Actual area value is from the North Aral Sea, because North Aral Sea is the smallest of the five study cases, and it is easiest to obtain its area by mapping two senses of Landsat images. Then, the relative error between the estimated area and the mapped actual area is calculated to evaluate the performance of area reconstruction. The relative error is calculated as follows:

(5) $$\delta =\frac{\left|E-A\right|}{A}\times 100\%$$

where δ, E, A are the mean value of relative error, estimated area and actual area.

### 4.2. Reconstructing Missing Lake Area and Water Level Records During 1984–2018

After reconstructing the lake area time series and evaluating the estimates, the months with both water level data and area data are picked out and a fitting formula for the unitary quadratic term of area and water level is established. The records of missing areas and water levels are reconstructed based on their empirical regression relationships. Eventually, monthly and annual average time series of lakes area and water level are derived for all the study period.

### 4.3. Reconstructing Time Series of Lake Volumes Variations from 1984 to 2018

We combined time series of lakes area and water level to further estimate the water volumetric changes of these lakes using a pyramid frustum volume estimation method. This method assumes the water body to be an inverted truncated pyramid [27]. It computes the change in volume at each time step with the change in the level and area of the truncated inverted pyramid with respect to the initial time step. In our study, the year in which each lake initially exists data is used as a baseline upon which values of the other years up to 2018 are compared. The cumulative summation of each time step volume change derived from the trapezoidal volume formula is given in equation:

(6) $$\Delta v=\frac{1}{3}\times \left(H_{t}-H_{0}\right)\times \left(A_{0}+A_{t}+\sqrt{\left(A_{0}\times A_{t}\right)}\right)$$

where:

Δv = Volume change with respect to the initial state (t0) at the nth month. *n* = Number of months;
H_t_ = Level of the water body at month t and H_0_ = Level of the water body at the previous month;
A_t_ = Area of the water extent at month t and A_0_ = Area of the water extent at the previous month.

## 5. Results and Discussions

### 5.1. Regression Analysis of Estimated Lake Area and Water Level

**Figure 3:**
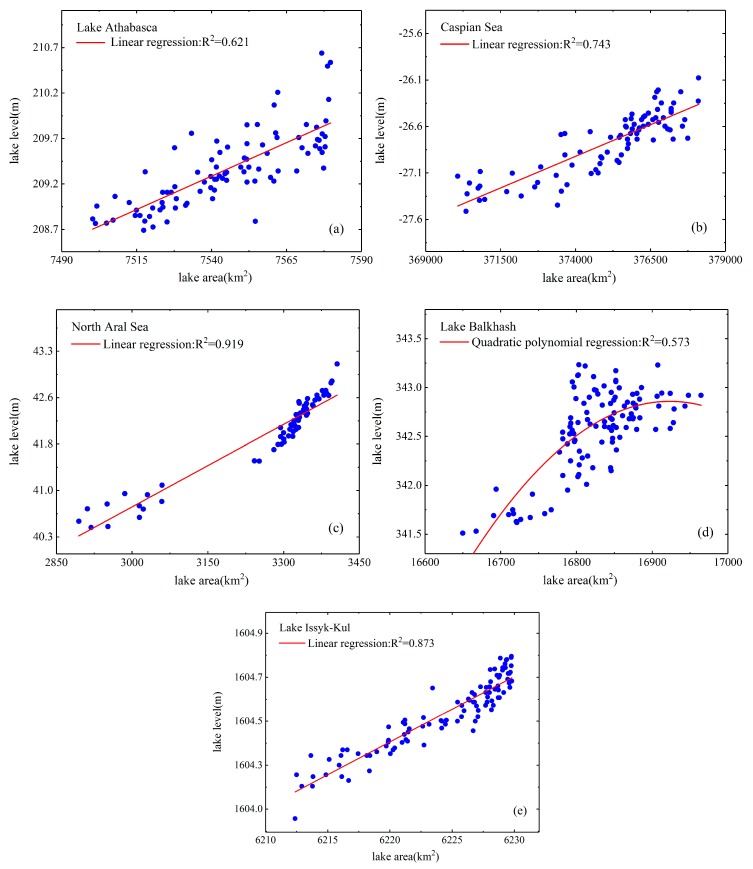
Area and water level regressions for Lake Athabasca (**a**), Caspian Sea (**b**), North Aral Sea (**c**), Lake Balkhash (**d**) and Lake Issyk-Kul (**e**), with R^2^ values of 0.621, 0.743, 0.919, 0.573 and 0.873 respectively.

In this part, the regression relationship of estimated lake area and water level series is analyzed. From Figure 3, the strong correlations between reconstructed inundation areas and water levels within the same observation month can be found for all of the five cases: the R^2^ values of Lake Athabasca, Caspian Sea, North Aral Sea, Lake Balkhash and Lake Issyk-Kul are 0.621, 0.743, 0.919, 0.573 and 0.873, respectively. The relatively weaker correlation was observed for Lake Balkhash, which may be mostly caused by the relatively less significant water frequency shifts over the selected lakeshore target area. In contrast, North Aral Sea whose terrain is flat at the extracted shoreline series, has the highest R^2^ value of 0.919 as its lake area changes more sharply with the water level than other lakes. The mean R^2^ of all the five lakes is 0.746, which indicates that the proposed method can produce the robust estimates of area time series for these large lakes.

### 5.2. Evaluation of Methods for Reconstructing Time Series of Area, Water Level and Volume Variation

For the accuracy of lake area reconstruction, we calculated the standard deviation of water frequency when deriving the averaged frequency of shoreline segment. It indicates the degree of dispersion of the water frequency fluctuation values by overlapping the shoreline with occurrence map. Then we further estimated the fluctuation range and uncertainties of lake volume variation based on the standard deviation of reconstructed lake area. The results show Lake Athabasca, Caspian Sea, North Aral Sea, Lake Balkhash and Lake Issyk-Kul five lakes have area variation ranges of 7336.08–7643.58 km^2^, 365,910.59–381,744.40 km^2^, 2745.80–3522.56 km^2^, 16,522.57–17,062.79 km^2^ and 6192.24–6234.32 km^2^, respectively, during the period of 1984–2018.

**Figure 4:**
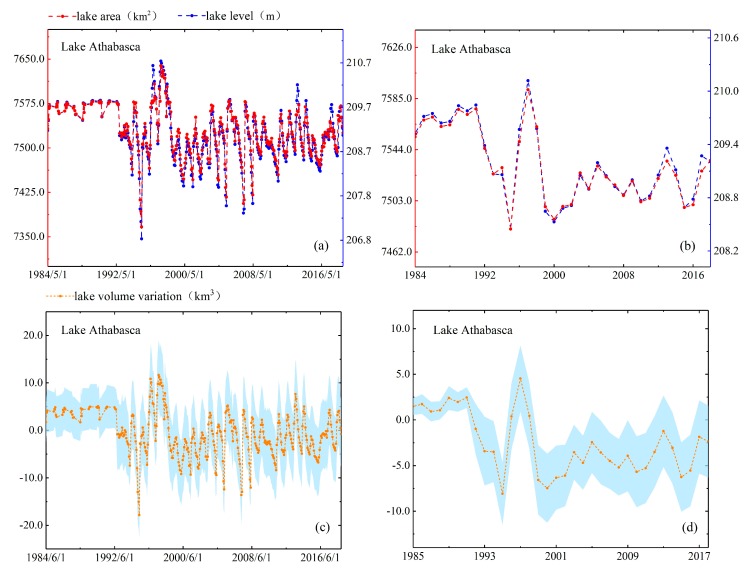
Lake area, level and volume variations in monthly (**a**,**c**) and annual (**b**,**d**) timescales for Lake Athabasca.

**Figure 5:**
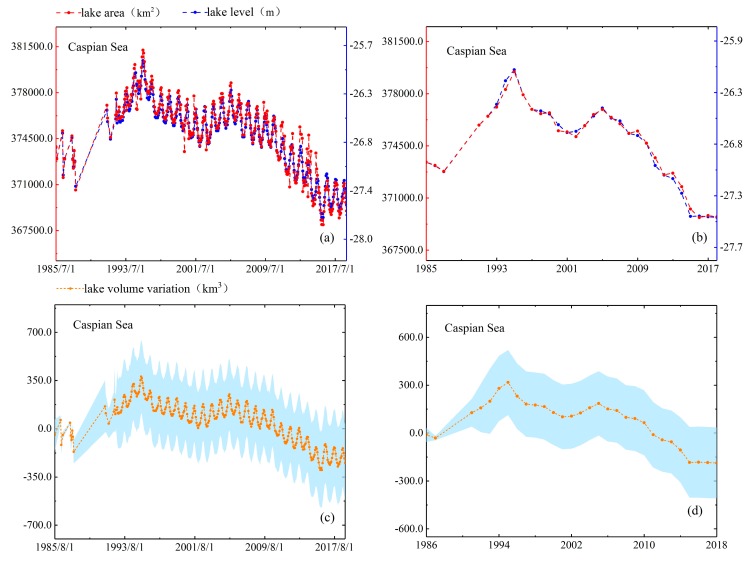
Lake area, level and volume variations in monthly (**a**,**c**) and annual (**b**,**d**) timescales for Caspian Sea.

**Figure 6:**
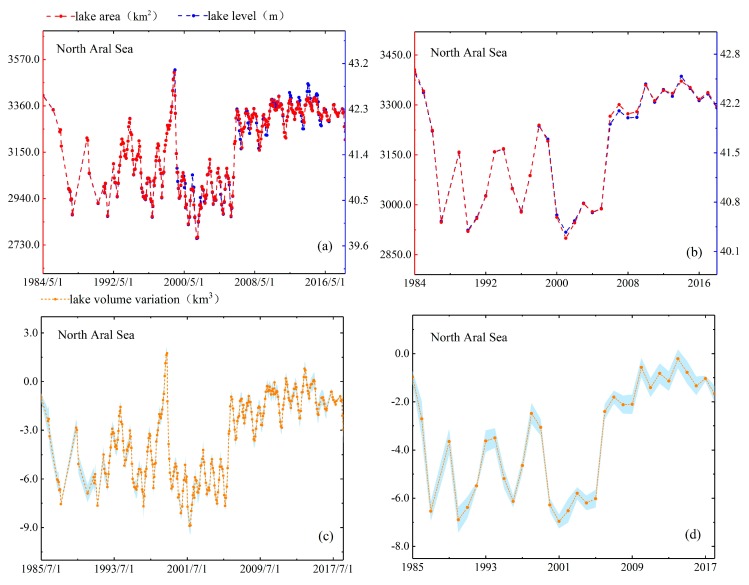
Lake area, level and volume variations in monthly (**a**,**c**) and annual (**b**,**d**) timescales for North Aral Sea.

**Figure 7:**
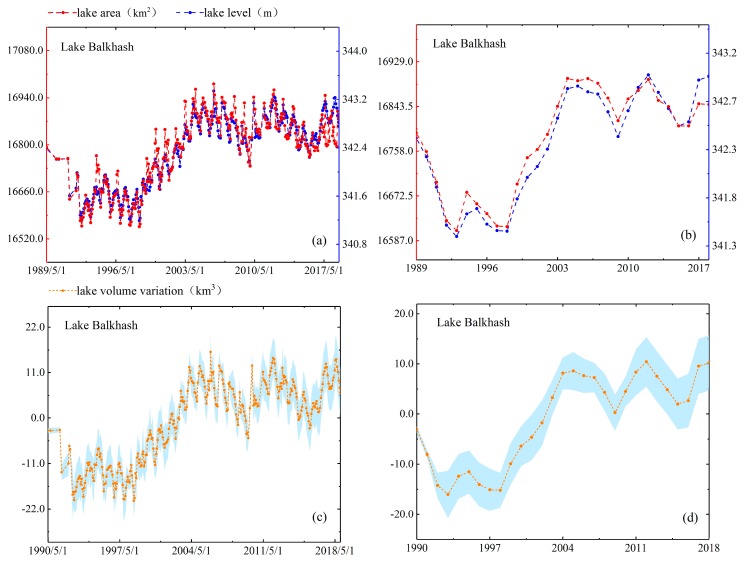
Lake area, level and volume variations in monthly (**a**,**c**) and annual (**b**,**d**) timescales for Lake Balkhash.

**Figure 8:**
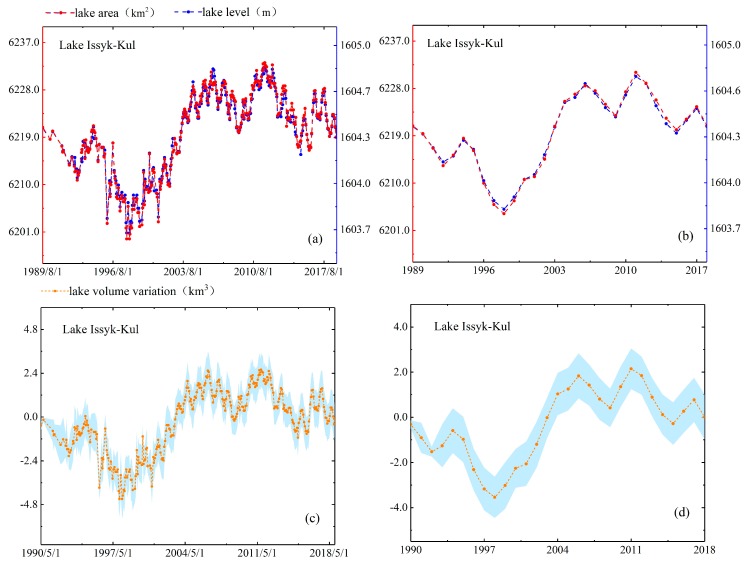
Lake area, level and volume variations in monthly (**a**,**c**) and annual (**b**,**d**) timescales for Lake Issyk-Kul.

As mentioned in Section 4.1.4, we use two methods to evaluate the performance of lake area estimates. In the first method, we compare estimated area with actual mapping area directly. North Aral Sea is the smallest lake among the five lakes and it has largest variation which the area change rate reached 24%. Moreover, the full mapping coverage of North Aral Sea only requires two Landsat scenes, so we obtain five area measurements of North Aral Sea in 2016–2018 directly based on the full inundation area mapping and compare them with the estimated areas. The results have shown that difference between the estimated areas and the actual areas of five observation dates is 46.05 km^2^ (in August 2016), 1.72 km^2^ (in May 2017), 20.69 km^2^ (in June 2018), 54.42 km^2^ (in July 2018) and 14.97 km^2^ (in July 2018). The values of relative error are 1.42%, 0.05%, 0.62%, 1.69% and 0.46%, respectively. In the second method, we check the consistency of their patterns by reconstructing the time series of area and water level. As shown in Figure 4, Figure 5, Figure 6, Figure 7 and Figure 8, we can conclude that there is a good agreement between the estimated area and water level, and both time series show consistent variations in the annual and seasonal timescales. It should be pointed out that Lake Athabasca does not show significant seasonality in 1984–1992 due to insufficient data coverage. Before 1992, there was neither water level data to derive the area, nor enough available images to map and estimate the lake area (thus the time series of volume variations is also lacked in 1984–1992).

### 5.3. Analysis of Lake Changes by Synthesizing the Reconstructed Area and Volume Variation Time Series and Prior Studies

#### 5.3.1. Lake Athabasca

Figure 4 presented the time series of Lake Athabasca showed overall decreasing trend in area during 1984–2018 with an obvious upward shift in 1996–1998 when the area was significantly higher than that in other years with the increase of 307.5 km^2^. Figure 4 illustrates that the maximum inundation area was observed in April 1995 to 7643.58 km^2^ and the minimum area was observed in July 1997 to 7336.08 km^2^. The reason why there was a sudden increase in the area was explained [28,29]. The study mentioned that the rise in water level was caused by flooding and the floods occurred in the summer of 1996 were achieved by the formation of ice jams. These results indicated our reconstruction of water level and area confirm the description of the 1996 water mass surge in published literature. It must be mentioned that the change profile of Lake Athabasca does not show remarkable seasonality in area, water level and volume between 1984 and 1992 due to the lack of adequate time series of satellite observations. The seasonal variations and trends of DAHITI water level in study of Schwatke et al. [30] agreed well with our results. As shown in Figure 4, the volume variation of Lake Athabasca ranged from +21.44 km^3^ (increased value) to –19.35 km^3^ (reduced value) and the months at which the maximum and minimum occurred coincided with the water level time series in 1996–1998.

#### 5.3.2. Caspian Sea

The time-series data from Figure 5 shows the area, water level and volume variation of Caspian Sea was generally decreasing with a nearly linear upward trend in 1987–1995 and 2001–2005, significant declines in 1995–2001 and 2005–2015 and relatively stable in 2015–2018. Worth noting that the maximum area occurred in June 1995 with a value of 381744.40 km^2^ and the minimum area occurred in February 2016 with a value of 365,910.59 km^2^ (see Figure 5). A possible explanation of these variations in areas could be largely attributed to hydrological condition changes of the Volga drainage basin [21]. For instance, Chen et al. [31] used climate model-predicted precipitation (P), evaporation (E), and observed river runoff (R) of Volga to reconstruct long-term Caspian Sea level (CSL) changes for 1979–2015. The conclusion rising surface temperatures had led to more evaporation in Caspian Sea than precipitation and runoff absorbed, leading to a reduction in area in recent decades was caused by the observed rapid CSL increase (about 12.74 cm/yr) and significant drop (~6.72 cm/yr) during the periods 1979–1995 and 1996–2015, respectively. At the same time, the annual cumulative evaporation rate of Caspian Sea will continue to increase in the foreseeable future as the Northern Hemisphere continues to warm up is pointed. In Ozyavas et al. [32], it is reported that the Volga River discharge in conjunction with evaporation over Caspian Sea were the two main components controlling water-level fluctuations of Caspian Sea. More specifically, Volga River discharge positively correlated with Caspian Sea water level rise and drop from 1993 to 1997 and in 1999, respectively. In 1997–2003, evaporation should dominate over the Volga River runoff and primarily controlled CSL fluctuations. As shown in Figure 5 the range of lake volume variation was shifted from +753.04 km^3^ (increased value) to −488.47 km^3^ (reduced value) which agree well with the overall downward trend in Figure 5. Another important factor attributed to the CS water-level decline is thought to be the transfer of CS water to the KBG bay through opening the dam in the KBG strait in 1995.

#### 5.3.3. North Aral Sea

We reconstructed the time-series data of North Aral Sea for the 35 years. Clearly to see, the change of lake area was not characterized by a monotonically increasing tendency. The evolution processes can be divided into three sub-stages: the first large-scale fluctuation in 1984–2005, the last small-scale fluctuation in 2006–2018 and the mid-sudden rise of water level in 2005–2006, which brought about increase of the average water level (in Figure 6). More specifically, the average water level of last period was 1.13 m higher than the first. In 1984–2018, the maximum water level appeared in April 1999 with a high lake stage of 43.11 m and the minimum water level (39.71 m) appeared in October 2001 (seen in Figure 6). Kouraev et al. [33] pointed out construction and destruction of several dams between the two parts of the lake attributed to the level of the North Aral Sea oscillated between of 39 m and 42 m and other research data confirmed this view. For example, Létolle and Chesterikoff [34] found that dams have been established to isolate them and keep the level of North Aral Sea since the separation of North Aral Sea and the South Aral Sea in 1989. In July 1992, the water pressure washed away the first 1-m- height sand dam. Then the second, third, fourth dams were built one by one which all were collapsed by flood in 1992–1999. The peak water level of North Aral Sea reached in April 1999 and then fell by 2.50 m at September 1999 both due to these incidents. The water level in North Aral Sea in 2005–2006 had risen markedly and had remained steadily rising since then attributed to the construction of fifth dam in August 2005. Kouraev et al. [33] analyzed the evolution of North Aral Sea based on radar altimeter data from T/P, Jason-1, ENVISAT and GFO satellites for 1992–2006. The water level changes presented in this study showed a similar pattern as average water level from our study which increased from 41.04 m in 1984–2005 to 42.17 m in 2006–2018. Moreover, the range of volume variation in North Aral Sea is +2.11 km^3^ (increased value) and –9.40 km^3^ (reduced value) could be observed in Figure 6 by reconstructing time series of volume variation. These discussions above confirm our work in analysis reasons for water level change, and the novel method can be effectively applicable in other lakes whose changes are similar with Northern Aral Sea.

#### 5.3.4. Lake Balkhash

As shown in Figure 7, Lake Balkhash presented a unique change pattern of area, water level and volume variation during 1989–2018. During the three decades, the tendency can be divided into four sub-periods (1994–1995, 1999–2006, 2010–2013 and 2016–2018) when lake levels showed upward shifts and four sub-periods (1989–1993, 1995–1999, 2006–2010 and 2013–2016) when lake levels showed downward shifts. Figure 7 showed the maximum area of Lake Balkhash was 17,062.79 km^2^ in March 2006 and the minimum area was 16,522.57 km^2^ in September 1998. Moreover, the volume variation of Lake Balkhash ranged from +19.21 km^3^ (increased value) to −25.60 km^3^ (reduced value) in Figure 7. For this, Bai et al. [35] proposed reservoirs and dams were two human factors that affect lake total runoff and lake area changes based on analyzing the surface area changes of nine major lakes from 1975 to 2007. As we all know, Lake Balkhash is mainly supplied by the Ili River, which covers 73% of the total inflow of water. Since 1974, artificial runoff had been controlled through the filling of the Kappa Gai Dam, and the annual flow of Ili River into Barhash has fallen to 77% of the mean before 1969 [36] which led water level and area to drop dramatically. Chao et al. [37] mentioned that there was a Nino Phenomenon from 1997 to 1998, which may cause the steep increase of water level in Lake Balkhash from 1998 to 2007. Propastin [38], who agreed with views from Chao et al. [37], made a point that the overall increase in precipitation and temperature in the catchment basin resulted in the water level of Lake Balkhash increased during 1998–2007. The temperature changes above Lake Balkhash could also support this view. The annual mean temperature over Lake Balkhash varied from 6.0 °C in 1976–1992 to 6.7 °C in 1998–2007. The temperature of this change directly led to a rise in water levels after 1998. These prior research findings can be well revealed in our reconstructed lake change series that area, water level and volume variations all have sharp rises after 1998.

#### 5.3.5. Lake Issyk-Kul

Interestingly, the water level curves for both Lake Issyk-Kul and Lake Balkhash indicated that not only the general trends derived from two time series were similar but that the abrupt changes and intra-annual variations of area, water level and volume variation were basically consistent in Figure 8. For instance, the time series of Lake Issyk-Kul showed overall upward shifts in area, water level and volume variation in 1989–2018. Moreover, water level of Lake Issyk-Kul declining in 1994–1998 and rising in 1998–2006 also agreed well with it in Lake Balkhash. We suspect this is cause they both in Central Asia with Lake Issyk-Kul is locate in south of Lake Balkhash with the nearest distance less than 300 km. Moreover, we speculate the Nino phenomenon may affect a wider area of this region. These geographical characteristics led them to share the same climate change which dominates the lake variations. Specific changes of Lake Issyk-Kul showed that the maximum area was 6234.32 km^2^ in August 2006 and the minimum area was 6192.24 km^2^ in March 1998 (see Figure 8). Besides, the volume variation ranged from +3.64 km^3^ (increased value) to −5.48 km^3^ (reduced value) could be seen from Figure 8. Song et al. [22] pointed out that the annual mean water level change has a strong correlation with precipitation and evaporation, but there may be no obvious correlation with the melting of glaciers caused by global warming. They also pointed out that water level and inundation area of Lake Issyk-Kul had piece-wise fluctuations, which largely agree with the periodical variations of precipitation [39]. In Klerx and Imanackunov [40], the water level of Lake Issyk-Kul had been dropping at a rate of about 4 cm/yr before 1998 and the water level has increased significantly at a rate of 10 cm/yr in the past eight years. Thus, they concluded this increase perhaps may be also associated with the recent acceleration of melting of glaciers associated with regional warming.

## 6. Summary

The inland lakes play a crucial role in global water cycle and sustain the supply of water resources. Continuous monitoring of these lakes is in strong demand. Difficulty of collecting spatially complete and temporally simultaneous remote sensing images, hard to guarantee image quality, and heavy labor and computing workload make it very difficult to map the large lakes. Therefore, a novel approach of reconstructing long-term and high-frequency time series of inundation areas of large lakes is proposed in this study. The general idea of this method is to obtain the lake inundation area at one specific observation date by referring to the mapping relationship of the WOF of selected shoreline segment and lake areas in the pre-established lookup table. The lookup table to map the links of the WOF and lake areas is derived from the JRC GSW dataset accessed in GEE. We select five large lakes worldwide to reconstruct the long time series (1984–2018) of inundation areas using this method, and to further compare with water level series and to model lake water volume variations. The results based on the case of North Aral Sea show that the mean relative error between estimated area and actual mapped value is about 0.85%. The reconstructed lake area changes also agree well with water level variations measured by satellite altimetry with the mean R^2^ of 0.746. In Supplementary Information, we did cases of the five lakes to map the changes in area over the past 35 years (1984–2018). These supplementary videos include Video S1 (Caspian Sea), Video S2 (Lake Athabasca), Video S3 (Lake Balkhash), Video S4 (Lake Issyk-Kul) and Video S5 (North Aral Sea). The results show that the correlation between water level and reconstructed area is closely related to flatness of lake shorelines at the selected target area. This research sheds new light on mapping large lakes at evidently deducted time and labor costs and be effectively applicable in other large lakes in regional and global scales. However, the novel method may not be applicable to the following scenarios: 1) Lakes expansion or shrinkage extent has exceeded the historical inundation fluctuation range of the water occurrence map of JRC GSW data set. Since the area-frequency lookup table is derived from WOF from 1984 to 2015, it is not possible to estimate the area for other years where the value is not between the maximum and minimum values. Thus, this method is not applicable to lakes with continued shrinkage such as South Aral Sea. 2) Lakes change dramatically or has no obvious changes. For example, Lake Victoria has steep shoreline terrains and thus show few areal changes during the study period.

## Figures and Tables

**Table 1:** Description of selected Landsat imagery scenes of the five lakes including: Lake Athabasca, Caspian Sea, North Aral Sea, Lake Balkhash and Lake Issyk-Kul.

Lake Name	Latitude Range	Longitude Range	Landsat Path/Row
Lake Athabasca	58°35′31′′ N–59°37′37′′ N	106°30′29′′ W–111°11′59′′ W	40/19, 41/18, 41/19
Caspian Sea	36°34′05′′ N–47°08′51′′ N	46°43′58′′E–54°04′21′′E	166/28
North Aral Sea	46°07′20′′ N–46°47′08′′ N	59°58′34′′E–61°32′51′′E	161/27, 161/28, 160/28
Lake Balkhash	45°02′54′′ N–46°50′04′′ N	73°23′48′′E–79°14′54′′E	149/27, 149/28, 148/28
Lake Issyk-Kul	42°09′04′′ N–42°46′16′′ N	76°10′58′′E–78°19′41′′E	149/30

**Table 2:** Details of different satellite altimetry missions.

Satellite	Altimeter	Revisit Cycle (Days)	Along-Track Spacing	Cross-Track Space (at the Equator)	Operational
Topex/Poseidon	Poseidon	10	~600 m–7 km	350 km	1992–2006
GFO	GFO-RA	17	~7 km	170 km	1998–2008
ENVISAT	RA2	30/35	~390 m–7 km	80 km	2002–2012
Jason-1	Poseidon-2	10	~300 m–7 km	350 km	2001–2013
Jason-2	Poseidon-3	10	~300 m–7 km	350 km	2008–
Jason-3	Poseidon-3B	10	~300 m–7 km	350 km	2015–
SARAL	AltiKa	17	~175 m	75 km	2013–
Sentinel-3A	SRAL	27	~300 m	104 km	2015–

**Table 3:** Water surface height from satellite altimetry and track number of satellite altimetry.

Lake Name	Water Surface Height from Satellite Altimetry & Track Number
Lake Athabasca	Topex/Poseidon (95/171/178/254), Jason-1,2,3 (95/178/254), GFO (93/142/179/228), ENVISAT (37/140/226/323/409/495/ 598/684/867/953), SARAL (140/226/409/495/598/684/867/953), Sentinel-3A (54/265/493/607/710)
Caspian Sea	Topex/Poseidon (16/31/57/92/107/133/168), Jason-1,2,3(16/31/57/92/133/168/ 209/244), GFO (42/53/70/128/139/214/225/300/311/386/397/444/455/472/483), ENVISAT (3/12/25/98/139/184/225/270/311/356/397/470/483/556/597/642/683/728/769/ 814/855/900/928/941), SARAL (98/184/225/270/311/397/483/556/642/728/769/855/928/941), Sentinel-3A (12/126/139/167/212/240/ 253/326/354/367/ 440/481/554/595/668/709)
North Aral Sea	Topex/Poseidon (107/218), Jason-1,2(107/218), ENVISAT (126/167/625)
Lake Balkhash	Topex/Poseidon (55/90/166/233/242), Jason-1,2,3(55/90/166/233), ENVISAT (51/96/137/182/223/268/309/395/554/595/640/681/726/767/812/853), SARAL (51/96/137/182/223/268/309/395/554/595/681/726/767/853), Sentinel-3A(51/96/165/210/279/324/365/479/552/593/666/707)
Lake Issyk-Kul	Topex/Poseidon (131), Jason-1,2,3(131), ENVISAT (10/223/554/767), SARAL (10/223/554/767), Sentinel-3A (10/593/666/707)

## Supplementary Materials

The following are available online at https://www.mdpi.com/1424-8220/19/19/4247/s1 and https://zenodo.org/record/3457606#.XYggPWYRVPY, Figure S1: Area-frequency correspondence table graded at intervals of 2.5% (a), 5% (b), 7.5% (c) and a summary of the three levels., Table S1: Relative error of estimated area and actual area under 2.5%, 5% and 7.5 intervals in North Aral Sea, Table S2: The specific year and month of the monthly lake area data via estimating with the lookup table, Video S1: Caspian Sea, Video S2: Lake Athabasca, Video S3: Lake Balkhash, Video S4: Lake Issyk-Kul, Video S5: North Aral Sea.
