# Supplementary material for: An Effective Low-Cost Remote Sensing Approach to Reconstruct the Long-Term and Dense Time Series of Area and Storage Variations for Large Lakes
Source: Sensors (Basel). 2019 Sep 30;19(19):4247. doi: 10.3390/s19194247 (PMC6806627; doi:10.3390/s19194247)
Supplement: Supplementary file 1 [file sensors-19-04247-s001.pdf]

Supplementary Information

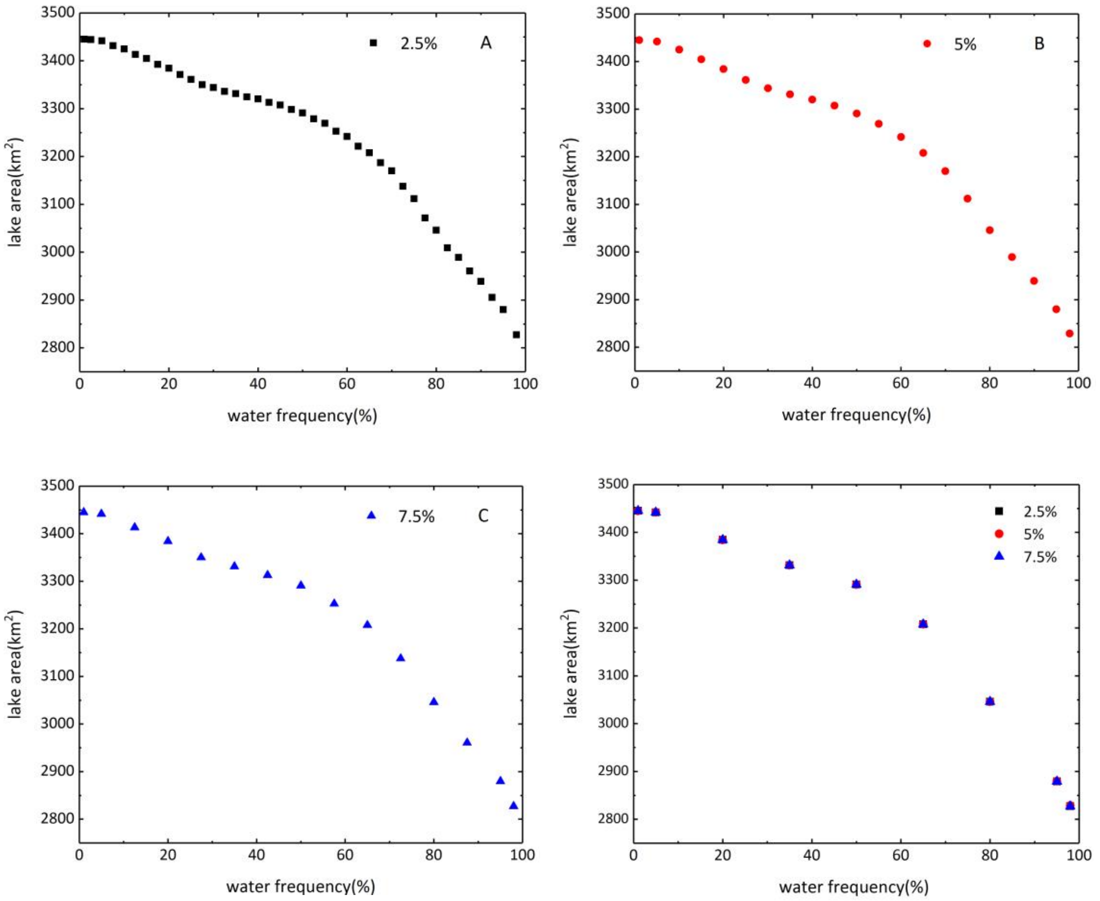

Figure SI. Area-frequency correspondence table graded at intervals of 2.5% (**a**), 5% (b), 7.5% (c) and a summary of the three levels.

Table SI. Relative error of estimated area and actual area under 2.5%, 5% and 7.5 intervals in North Aral Sea.

Grading	Area (2.5%)	Area (5%)	Area (7.5%)	Relative Error 2.5%&5%	Relative Error 7.5%&5%
98%	2827.17	2828.80	2827.17	-0.0578%	-0.0570%
95%	2878.98	2879.98	2878.98	-0.0340%	-0.0340%
80%	3045.88	3045.89	3045.88	-0.0002%	-0.0002%
65%	3207.94	3207.94	3207.94	-0.0001%	-0.0001%
50%	3290.98	3290.98	3290.98	-0.0001%	-0.0001%
35%	3331.17	3331.18	3331.17	-0.0002%	-0.0002%
20%	3384.38	3384.38	3384.38	-0.0001%	-0.0001%
5%	3441.73	3441.73	3441.73	-0.0002%	-0.0002%
1%	3444.92	3444.93	3444.92	-0.0001%	-0.0001%

Table SI. The specific year and month of the monthly lake area data via estimating with the lookup table.

A. Lake Athabasca

Year	1	2	3	4	5	6	7	8	9	10	11	12
1984					■	■	■	■				
1985					■	■	■	■	■			
1986					■	■	■	■	■			
1987					■	■	■	■	■			
1988						■	■	■	■			
1989						■	■	■				
1990					■	■	■	■	■			
1991						■	■	■				
1992						■	■	■				
1993							■	■	■			
1994					■		■	■	■			
1995							■		■			
1996					■	■	■	■	■			
1997						■						
1998					■	■	■	■	■			
1999						■	■	■	■			
2000							■	■	■			
2001						■	■	■	■			
2002						■	■	■	■			
2003					■	■		■				
2004						■	■	■	■			
2005						■	■		■			
2006					■		■	■				
2007					■	■	■	■	■			
2008					■	■		■				
2009						■	■	■	■			
2010						■	■	■				
2011							■	■	■			
2012									■			
2013					■	■	■	■		■		
2014					■	■	■	■	■			
2015				■	■	■		■				
2016						■		■	■			
2017						■	■	■	■			
2018				■		■						

B. The Caspian Sea

Year	1	2	3	4	5	6	7	8	9	10	11	12
1984												
1985							■	■				
1986				■	■		■					
1987					■	■	■	■	■	■		
1988												
1989												
1990												
1991					■	■				■		
1992					■	■	■	■				
1993						■	■					
1994								■	■	■		
1995			■									
1996												
1997												
1998				■			■	■	■			
1999										■		
2000			■	■	■	■		■	■			
2001				■			■	■	■			
2002				■		■	■		■			
2003				■								
2004				■								
2005				■				■				
2006							■	■	■	■		
2007						■	■	■	■			
2008					■	■	■					
2009				■		■	■	■	■	■		
2010					■	■	■	■	■			
2011				■	■	■	■		■			
2012				■	■		■	■	■			
2013					■	■	■	■	■	■		
2014			■	■	■	■	■	■	■			
2015				■	■	■	■	■	■			
2016						■	■					
2017									■			
2018												

C. The North Aral Sea

Year	1	2	3	4	5	6	7	8	9	10	11	12
1984					■							
1985							■					
1986				■	■	■						
1987				■	■	■	■	■	■			
1988												
1989					■	■		■				
1990								■				
1991				■	■	■			■			
1992					■	■	■	■				
1993												
1994												
1995												
1996												
1997												
1998												
1999				■			■	■				
2000				■	■	■	■					
2001				■		■						
2002			■					■	■			
2003				■								
2004						■						
2005						■						
2006				■		■	■	■	■	■		
2007					■	■	■	■	■	■		
2008				■			■			■		
2009				■	■			■	■			
2010				■		■		■	■	■		
2011							■					
2012				■	■	■				■		
2013				■	■	■	■	■	■	■	■	
2014				■	■	■	■	■	■	■		
2015				■	■	■	■	■	■	■	■	
2016			■	■	■				■	■		
2017				■	■	■	■		■	■	■	
2018				■	■	■	■	■				

D. Lake Balkhash

Year	1	2	3	4	5	6	7	8	9	10	11	12
1984												
1985												
1986												
1987												
1988												
1989					■							
1990					■			■				
1991							■		■			
1992					■	■	■					
1993				■								
1994					■	■	■	■	■			
1995												
1996						■	■					
1997												
1998												
1999						■		■	■	■		
2000				■	■		■	■		■		
2001				■	■		■	■	■			
2002					■		■	■	■	■		
2003				■	■							
2004					■					■		
2005						■		■	■			
2006				■			■	■	■	■		
2007					■	■	■	■	■	■		
2008				■	■	■	■	■	■			
2009				■		■				■		
2010				■	■	■	■	■	■	■		
2011					■	■	■	■	■	■		
2012					■	■		■	■	■		
2013					■	■	■	■	■	■		
2014			■	■	■	■	■	■	■	■		
2015					■	■	■	■	■	■		
2016				■	■	■	■	■	■			
2017			■	■	■		■	■	■		■	
2018			■	■	■	■	■	■				

E. Lake Issyk-Kul

Year	1	2	3	4	5	6	7	8	9	10	11	12
1984												
1985												
1986												
1987												
1988												
1989								■				
1990					■			■				
1991							■	■	■			
1992				■			■			■		
1993						■	■	■	■			
1994					■	■	■	■	■	■		
1995										■		
1996				■								
1997												
1998												
1999												
2000									■			
2001								■	■			
2002				■	■	■	■	■		■		
2003				■	■							
2004					■	■	■	■		■		
2005				■						■		
2006						■	■	■	■	■		
2007					■	■	■	■	■	■		
2008				■	■	■	■	■	■	■		
2009					■	■	■	■	■			
2010				■	■	■	■	■	■	■		
2011					■	■	■	■	■			
2012				■			■	■		■		
2013				■	■	■	■	■	■	■	■	
2014				■	■	■	■	■	■	■		
2015				■	■	■	■	■	■	■		
2016												
2017				■	■		■	■	■		■	
2018			■			■	■	■				
